# Supplementary figures and images for: Blood pressure changes during the first 24 hours of life and the association with the persistence of a patent ductus arteriosus and occurrence of intraventricular haemorrhage
Source: PLoS One. 2021 Nov 30;16(11):e0260377. doi: 10.1371/journal.pone.0260377 (PMC8631614; doi:10.1371/journal.pone.0260377)

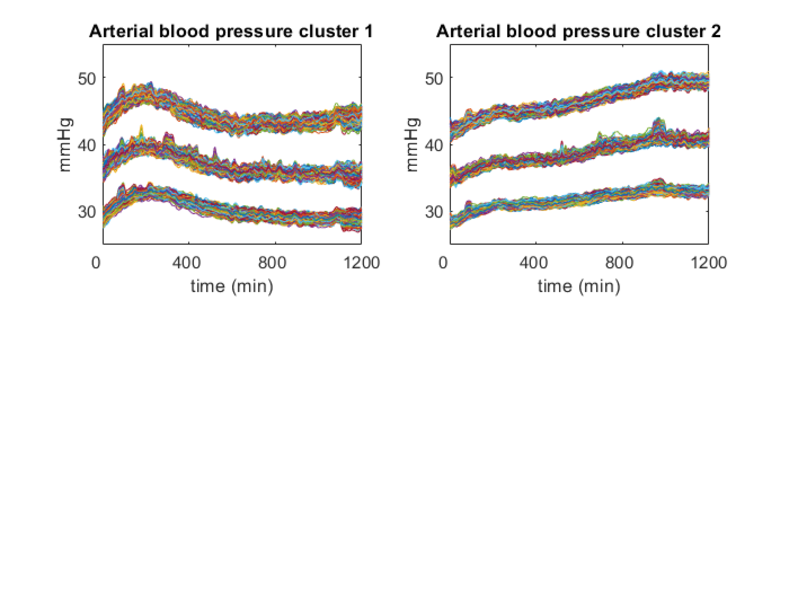

Supplement: S1 Fig — We repeated the k-means clustering analysis 4000 times with bootstrap samples to examine the clustering stability. The graph overlays the mean arterially recorded systolic, mean, and diastolic blood pressure measurements from each of the 4000 clustering analyses done with the bootstrap samples. (TIF) [file pone.0260377.s001.tif]

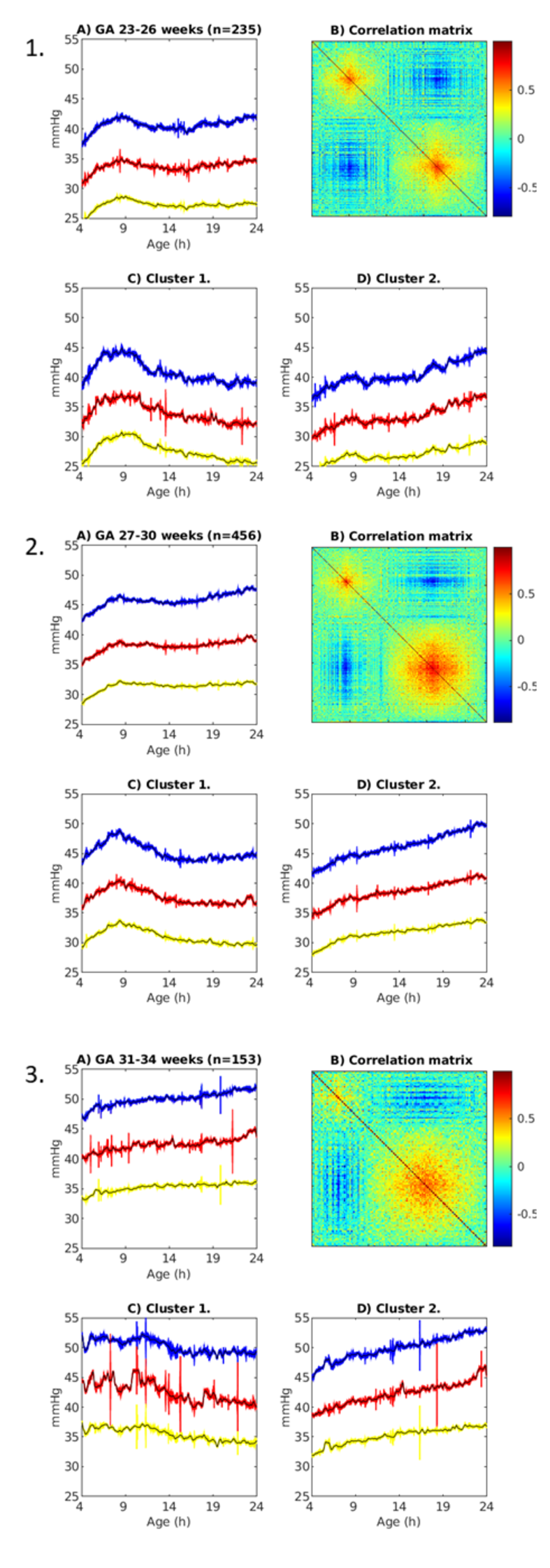

Supplement: S2 Fig — (A) The mean and the standard error for the systolic (blue), mean (red), and diastolic (yellow) arterially recorded blood pressure during the first 4–24 hours after birth. (B) The correlation matrix shows how k-means clustering divided the infants into two clusters based on how similar the infants’ blood pressure time series were. (C) Arterially recorded blood pressure for infants in cluster 1. (D) Arterially recorded blood pressure for infants in cluster 2. Data are shown separately for infants with gestational age (GA) of (1.) 23–26 weeks, (2.) 27–30 weeks and (3.) 31–34 weeks. Infants with lower mean MAP at 18–24 hours than at 4–10 hours after birth and a GA of 23–26 weeks had a trend for an increased occurrence of IVH of grades III-IV (24% vs 14%, odds ratio = 1.97; CI 1–3.86; p = 0.059; Fischer’s exact test). Infants with lower mean MAP at 18–24 hours than at 4–10 hours after birth and a gestational age of 27–30 weeks had an increased likelihood of needing surgical PDA interventions after pharmacological PDA interventions (9% vs 2%, odds ratio = 4.43; CI 1.7–11.56; p = 0.001; Fischer’s exact test), and a trend for an increased occurrence of IVH of grades I–II (12% vs 7%, odds ratio = 1.8; CI 0.94–3.46; p = 0.09; Fischer’s exact test) and IVH of grades III–IV (8% vs 4%, odds ratio = 2.27; CI 0.98–5.22; p = 0.055; Fischer’s exact test). Infants with lower mean MAP at 18–24 hours than at 4–10 hours after birth and gestational age of 31–34 weeks had a trend for an increased occurrence of IVH of grades III-IV (4% vs 0%, odds ratio = NA; CI NA; p = 0.089; Fischer’s exact test). (TIF) [file pone.0260377.s002.tif]
