# Supplementary material for: Blood pressure changes during the first 24 hours of life and the association with the persistence of a patent ductus arteriosus and occurrence of intraventricular haemorrhage
Source: PLoS One. 2021 Nov 30;16(11):e0260377. doi: 10.1371/journal.pone.0260377 (PMC8631614; doi:10.1371/journal.pone.0260377)
Supplement: S1 Table — The table shows the odds ratio, confidence intervals and p-values for each variable. The table also shows the number and percentage of infants who had surgical PDA treatment according to the binary definition provided by each grouping variable. Patent ductus arteriosus (PDA), Small for gestational age (SGA), respiratory distress syndrome (RDS), confidence interval (CI), mean arterial blood pressure (MAP). (DOCX) [file pone.0260377.s003.docx]

**S1 Table. The multinominal logistic regression analysis results for variables predicting surgical interventions of a patent ductus arteriosus.**

|  | No surgical treatment (n = 705) vs surgical treatment (n = 139) | | | |
| --- | --- | --- | --- | --- |
|  | Odds ratio | n_yes_ (%) / n_no_ (%) | 95 CI for odds ratio | p-value |
| Gestational age (< 28 weeks, yes/no) | 6.34 | 121 (35) / 18 (4) | (3.45–11.64) | p < 0.001 |
| SGA (yes/no) | 0.91 | 21 (10) / 118 (19) | (0.5–1.66) | p = 0.753 |
| RDS (yes/no) | 1.37 | 95 (23) / 44 (10) | (0.86–2.17) | p = 0.181 |
| Sepsis (yes/no) | 0.68 | 27 (18) / 112 (16) | (0.4–1.14) | p = 0.144 |
| Antenatal corticosteroids (yes/no) | 2.11 | 134 (17) / 5 (14) | (0.75–5.92) | p = 0.155 |
| Fluid > 120 ml/kg (yes/no) | 2.04 | 107 (26) / 32 (7) | (1.25–3.33) | p = 0.004 |
| MAP18–24 < MAP4–10 hours after birth (Group 1, yes/no) | 1.45 | 74 (23) / 65 (13) | (0.95–2.2) | p = 0.086 |
| MAP < 33 mmHg (yes/no) | 1.24 | 61 (37) / 78 (11) | (0.78–1.97) | p = 0.362 |
| Inotrope (yes/no) | 1.18 | 101 (24) / 38 (9) | (0.72–1.91) | p = 0.511 |
| Invasive ventilation (yes/no) | 2.07 | 133 (25) / 6 (2) | (0.71–6.05) | p = 0.182 |
| Surfactant administration (yes/no) | 3.45 | 137 (22) / 2 (1) | (0.62–19.11) | p = 0.157 |

The table shows the odds ratio, confidence intervals and p-values for each variable. The table also shows the number and percentage of infants who had surgical PDA treatment according to the binary definition provided by each grouping variable. Patent ductus arteriosus (PDA), Small for gestational age (SGA), respiratory distress syndrome (RDS), confidence interval (CI), mean arterial blood pressure (MAP).
