# Supplementary material for: Blood pressure changes during the first 24 hours of life and the association with the persistence of a patent ductus arteriosus and occurrence of intraventricular haemorrhage
Source: PLoS One. 2021 Nov 30;16(11):e0260377. doi: 10.1371/journal.pone.0260377 (PMC8631614; doi:10.1371/journal.pone.0260377)
Supplement: S2 Table — The first column includes estimates for the odds of having no IVH vs having IVH of any grade. The second column includes estimates for the odds of having a grade I–II IVH versus a grade III–IV IVH, given that the infant had an IVH. The table shows the odds ratio, confidence intervals and p-values for each variable. The table also shows the number and percentage of infants who had IVH of grade I–II (third column) and IVH of grade III–IV (seventh column) according to the binary definition provided by each grouping variable. Small for gestational age (SGA), respiratory distress syndrome (RDS), confidence interval (CI), mean arterial blood pressure (MAP). (DOCX) [file pone.0260377.s004.docx]

**S2 Table.** **The multinominal logistic regression analysis results for variables predicting the risk of having no intraventricular haemorrhage (IVH) and having grade I-II or III-IV IVH.**

|  | No IVH (n = 690) vs IVH (n = 142) | | | | Grade I-II IVH (n = 74) vs grade III-IV IVH (n = 68) | | | |
| --- | --- | --- | --- | --- | --- | --- | --- | --- |
|  | Odds ratio | nyes (%) / nno (%) | 95 CI for odds ratio | p-value | Odds ratio | nyes (%) / nno (%) | 95 CI for odds ratio | p-value |
| Gestational age (< 28 weeks, yes/no) | 2.67 | 47 (14) / 27 (5) | (1.59–4.48) | p < 0.001 | 0.83 | 51 (15) / 17 (3) | (0.31–2.22) | p = 0.705 |
| SGA (yes/no) | 0.37 | 7 (3) / 67 (11) | (0.2–0.69) | p = 0.002 | 1.61 | 7 (3) / 61 (10) | (0.45–5.8) | p = 0.466 |
| RDS (yes/no) | 1.57 | 47 (11) / 27 (6) | (1.01–2.46) | p = 0.046 | 1.25 | 48 (11) / 20 (5) | (0.55–2.84) | p = 0.597 |
| Sepsis (yes/no) | 0.74 | 14 (9) / 60 (9) | (0.45–1.23) | p = 0.241 | 0.94 | 11 (7) / 57 (8) | (0.36–2.46) | p = 0.898 |
| Antenatal corticosteroids (yes/no) | 1.08 | 71 (9) / 3 (8) | (0.46–2.54) | p = 0.866 | 0.39 | 63 (8) / 5 (14) | (0.07–2.04) | p = 0.264 |
| Fluid > 120 ml/kg (yes/no) | 1.79 | 45 (11) / 29 (7) | (1.14–2.79) | p = 0.011 | 2.37 | 53 (13) / 15 (3) | (0.98–5.77) | p = 0.056 |
| MAP18–24 < MAP4–10 hours after birth (Group 1, yes/no) | 1.83 | 38 (12) / 36 (7) | (1.23–2.72) | p =0.003 | 1 | 40 (12) / 28 (5) | (0.48–2.12) | p = 0.99 |
| MAP < 33 mmHg (yes/no) | 1.04 | 19 (12) / 55 (8) | (0.65–1.67) | p = 0.875 | 1.48 | 27 (17) / 41 (6) | (0.64–3.44) | p = 0.358 |
| Inotrope (yes/no) | 1.01 | 48 (11) / 26 (6) | (0.64–1.57) | p = 0.98 | 0.5 | 44 (11) / 24 (6) | (0.21–1.17) | p = 0.109 |
| Invasive ventilation (yes/no) | 0.7 | 55 (11) / 19 (6) | (0.35–1.4) | p = 0.308 | 4.27 | 61 (12) / 7 (2) | (0.98–18.6) | p = 0.053 |
| Surfactant administration (yes/no) | 2.07 | 66 (11) / 8 (3) | (0.84–5.1) | p = 0.116 | 0.73 | 65 (11) / 3 (1) | (0.1–5.28) | p = 0.755 |

The first column includes estimates for the odds of having no IVH vs having IVH of any grade. The second column includes estimates for the odds of having a grade I–II IVH versus a grade III–IV IVH, given that the infant had an IVH. The table shows the odds ratio, confidence intervals and p-values for each variable. The table also shows the number and percentage of infants who had IVH of grade I–II (third column) and IVH of grade III–IV (seventh column) according to the binary definition provided by each grouping variable. Small for gestational age (SGA), respiratory distress syndrome (RDS), confidence interval (CI), mean arterial blood pressure (MAP).
