# Supplementary material for: Blood pressure changes during the first 24 hours of life and the association with the persistence of a patent ductus arteriosus and occurrence of intraventricular haemorrhage
Source: PLoS One. 2021 Nov 30;16(11):e0260377. doi: 10.1371/journal.pone.0260377 (PMC8631614; doi:10.1371/journal.pone.0260377)
Supplement: Attachment — Submitted filename: Response_to_Reviewers.docx [file pone.0260377.s007.docx]

PONE-D-21-20256R1

Blood pressure changes during the first 24 hours of life and the association with the persistence of a patent ductus arteriosus and occurrence of intraventricular haemorrhage

PLOS ONE

Dear Dr. Boldt,

Thank you for submitting your manuscript to PLOS ONE. After careful consideration, we feel that it has merit but does not fully meet PLOS ONE's publication criteria as it currently stands. Therefore, we invite you to submit a revised version of the manuscript that addresses the points raised during the review process.

Both reviewers highly appreciate your efforts to address their points raised during the previous round of review. I kindly ask you to respond to the outstanding comments raised by reviewer 2.

Please submit your revised manuscript by Dec 09 2021 11:59PM. If you will need more time than this to complete your revisions, please reply to this message or contact the journal office at [plosone@plos.org](mailto:plosone@plos.org). When you're ready to submit your revision, log on to <https://www.editorialmanager.com/pone/> and select the 'Submissions Needing Revision' folder to locate your manuscript file.

Please include the following items when submitting your revised manuscript:

- A rebuttal letter that responds to each point raised by the academic editor and reviewer(s). You should upload this letter as a separate file labeled 'Response to Reviewers'.
- A marked-up copy of your manuscript that highlights changes made to the original version. You should upload this as a separate file labeled 'Revised Manuscript with Track Changes'.
- An unmarked version of your revised paper without tracked changes. You should upload this as a separate file labeled 'Manuscript'.

If you would like to make changes to your financial disclosure, please include your updated statement in your cover letter. Guidelines for resubmitting your figure files are available below the reviewer comments at the end of this letter.

If applicable, we recommend that you deposit your laboratory protocols in protocols.io to enhance the reproducibility of your results. Protocols.io assigns your protocol its own identifier (DOI) so that it can be cited independently in the future. For instructions see: <https://journals.plos.org/plosone/s/submission-guidelines#loc-laboratory-protocols>. Additionally, PLOS ONE offers an option for publishing peer-reviewed Lab Protocol articles, which describe protocols hosted on protocols.io. Read more information on sharing protocols at <https://plos.org/protocols?utm_medium=editorial-email&utm_source=authorletters&utm_campaign=protocols>.

We look forward to receiving your revised manuscript.

Kind regards,

Harald Ehrhardt
Academic Editor
PLOS ONE

*Dear Prof. Dr. med. Ehrhardt,*

*Thank you for allowing us to revise our manuscript. We want to thank the editors and the reviewers for their valuable comments. Furthermore, we thank reviewer 1 for previous valuable comments and your appreciation of our study.*

*Reviewer 2*

*We are grateful for the valuable insights you provided us during the major and minor revisions. Below, we address each comment raised during the latter review process.*

Reviewer #2: I would like to thank the Editor and the Authors for the opportunity to
re-review the manuscript entitled now: "Blood pressure
changes during the first 24 hours of life and the association with the persistence of a
patent ductus arteriosus and occurrence of intraventricular haemorrhage".

Thank you very much for responding to all comments and questions and for performing the requested analysis.

I have some questions. have you used in your cohort postnatal prophylactic corticosteroids?

*On page 19, lines 357-358 of the manuscript, we now state the following:*

*"Postnatal prophylactic steroids were not used in the patient cohort."*

I have some suggestions for a better visibility of your data:
Would it be possible to convert table 2 into 2 tables, it seems to me that there is a lot of information in table 2. One in relation to PDA and the other with HIV?

*Thank you for the suggestion. We now divided table 2 into two separate tables (Table 2 page 15 and Table 3 page 16).*

From my point of view I think it would be better to say that group 1 is the group that has had the lower mean MAP at 18-24h instead of saying that group 1 has had the higher mean MAP at 4-10h. For me it's much clearer and the message makes more sense, but it's just a personal opinion. It's up to you if you want to change this.

*To improve the reading experience, we decided to change the wording as proposed.*
